# A phantom mimicking layered biological microenvironments investigated with 7T diffusion weighted MRI

**DOI:** 10.1038/s41598-026-47120-6

**Published:** 2026-04-16

**Authors:** Łukasz Łabieniec, Krzysztof Szymański, Michał Wieteska, Grzegorz Domański, Marek Brancewicz, Łukasz Lisowski, Kamil Lipiński, Ryszard Łaźny, Piotr Bogorodzki

**Affiliations:** 1 Faculty of Physics, University of Bialystok, Bialystok, Poland; 2 Faculty of Electronics and Information Technology, Warsaw University of Technology, Warsaw, Poland; 3 Department of Ophthalmology, Medical University of Bialystok, Bialystok, Poland; 4 Faculty of Chemistry, University of Bialystok, Bialystok, Poland; 5 Polish Academy of Sciences (IMDIK PAN), Mossakowski Medical Research Institute, Warsaw, Poland

**Keywords:** Diffusion MRI, Spin-echo, Signal attenuation, Reflecting walls, Laminar diffusion, Anisotropic phantom, Biological physics, Chemical physics, Condensed-matter physics

## Abstract

We developed a physical phantom that mimics laminar biological microstructure with controlled geometric and diffusional properties. The primary goal of this study is to characterize the diffusion properties of the phantom and to assess how laminar microstructural geometry influences diffusion-weighted (DW) magnetic resonance imaging (MRI) signals. The phantom consists of stacked polyethylene foils forming water layers with thicknesses ranging from ~ 1 to 16 μm. Water diffusion within these layers is free in-plane but restricted perpendicular to the boundaries. We initially modeled the perpendicular component of the signal using a Laplacian spectral approach, while the parallel component was described by standard Gaussian attenuation. Although this model captures the overall signal behavior, the measured data reveal systematic deviations attributable to local structural inhomogeneities, such as the formation of water pockets between foil layers. To account for these effects, we introduce an extension of the model that incorporates spatial variations in layer spacing. Our data, acquired on a 7 T MRI scanner, show good agreement with the extended model and suggest the presence of such water pockets. Their planar dimensions, which range from 28 to 1500 μm, depend on foil compression and scale proportionally with the thickness of the water layer. These findings show that accurate diffusion models are needed to correctly estimate the phantom’s microstructure, and that the developed phantom is a useful tool for studying laminar diffusion. The remaining mismatch between the model and the data suggests that additional structural factors, not captured by the model, also influence the measurements.

**Supplementary Information:**

The online version contains supplementary material available at 10.1038/s41598-026-47120-6.

## Introduction

### Explanations

In this work, we refer to laminar biological microenvironments, i.e., structures composed of parallel layers that restrict molecular motion across layers. Unlike laminar flow, which describes an orderly fluid motion, our experiments focus on self-diffusion, where the mean molecular displacement is zero but diffusion is hindered by the layered geometry.

A laminar system, in our context, refers to a geometric arrangement of parallel structures (such as plates or layers) that restrict molecular motion perpendicular to them. This definition should not be confused with laminar flow; it pertains exclusively to the layered geometry itself and its influence on diffusion, independent of any bulk fluid movement.

### Background and motivation

Diffusion in layered biological structures is strongly shaped by geometric constraints, yet the relationship between laminar microstructure and diffusion-weighted (DW) magnetic resonance imaging (MRI) signals remains insufficiently understood. Several studies have noted that commonly used diffusion models are based on idealized assumptions that may not hold in systems with pronounced laminar organization^1–3^. Layered systems appear in many contexts, including cortical grey matter^2,4^, the optic nerve^5^, and model membranes^6^. A relevant example is the subarachnoid space of the human optic nerve, where arachnoid trabeculae and septa partition the space into compartments with thicknesses on the order of ~ 15 μm^7^, comparable to water layers in our phantom. These barriers influence cerebrospinal fluid diffusion along the nerve sheath.

While real tissues involve additional complexities—such as cellular shapes, membrane permeability, and heterogeneous composition—a simplified laminar system provides a controlled way to isolate the effects of geometric confinement. Layered geometries are also encountered in non-biological settings, such as fluid transport in rocks^8^, underscoring the broader relevance of such models.

In this study, we focus specifically on characterizing diffusion in a well-defined laminar phantom and evaluating how geometric features influence DW-MRI measurements. All theoretical modeling and analysis are directed toward this goal.

### The role of physical phantoms in diffusion weighted imaging

Physical phantoms provide a controllable means to test and refine theoretical models. Numerous phantom designs for diffusion imaging have been developed, differing in materials, geometry, and internal architecture. Properly designed phantoms offer a known reference for specific measured parameters, enabling the validation and optimization of experimental settings prior to in vivo studies. Commonly used DWI phantoms include spherical, cylindrical, fibrous, or tubular structures.

Early implementations employed well-defined geometries that allow characterization of restricted diffusion and anisotropy under controlled conditions. Yanasak et al.^9^ constructed DTI phantoms from water-filled arrays of glass capillaries, demonstrating reproducible fractional anisotropy (FA) and apparent diffusion coefficient (ADC) measurements across multiple phantoms. Other early studies used synthetic fibers: Kimura et al.^10^ employed ultra-high molecular weight polyethylene and high-performance polyethylene fibers packed in heat-shrinkable tubes to generate fiber phantoms with tunable fiber density (FD), while Hubbard et al.^11^ developed electrospun hollow fibers made of poly(ε-caprolactone) (PCL) with an inner polyethylene oxide (PEO) core, aligned and packed in layers inside glass tubes, with voids filled by cyclohexane to mimic axonal water diffusion.

Lin et al.^12^ designed a system of polytetrafluoroethylene (PTFE) tubes arranged in orthogonal sheets to simulate crossing fibers. Fan et al.^13^ proposed a multi-module diffusion phantom, where hollow polypropylene filaments produced by melt-spinning were arranged in parallel or crossing geometries using 3D-printed holders, allowing controlled variation of fiber packing density along the bundle length.

More recently, fully 3D-printed phantoms have been developed to achieve higher biomimicry and tunability. Mushtaha et al.^14^ printed phantoms from a composite material that, after dissolution of polyvinyl alcohol, exhibited microscopic fibrous pores, enabling assessment of diffusion kurtosis metrics and reproducibility between phantoms. These designs illustrate the trend towards modular and tunable architectures that mimic tissue microstructure more realistically.

It is worth noting that the vast majority of these phantoms were designed to study anisotropic diffusion. However, planar diffusion, which is relevant for layered biological structures, has also been addressed. The most comparable solution to our current study was proposed by Krzyżak et al.^15,16^, who constructed a laminar phantom consisting of thin glass plates (≈100 µm) separated by water layers (≈20 µm), arranged in a brick-like structure (2.5 cm × 2 cm) and immersed in a water-filled tube to provide a well-defined planar diffusion environment. While this design successfully reproduces layered anisotropic geometries, the brittleness of glass limits the minimum achievable water layer thickness and prevents graded compression or local adjustments of interlaminar spacing. These constraints motivate the development of more flexible laminar phantoms capable of simulating biologically relevant variations in layer thickness and geometry.

### A novel phantom design

Inspired by these limitations and building upon recent work on anomalous diffusion^1^ and lipid bilayer models^6^ in this study we developed a novel phantom composed of commercially available thin polyethylene foils for diffusion in laminar system. By adjusting compression, we control both the number and thickness of interleaved water layers, enabling the simulation of biologically relevant laminar constraints. The most comparable system to our work is the patented phantom^17–19^, composed of stacked glass plates with thicknesses of 180–200 μm, separated by water layers of 20–40 μm. While these constructs offer valuable insights into anisotropic diffusion, they suffer from rigidity—making it difficult to vary the interlaminar spacing or introduce microscopic irregularities.

### Description of restricted diffusion in a laminar system

Theoretical descriptions of free and anisotropic Gaussian diffusion are well established and extensively documented in the diffusion MRI literature^20–23^. In the present work, we therefore introduce only the notation required for the restricted-diffusion model considered below. The diffusion-weighted signal attenuation is expressed as

1 $$E=\frac{S(b)}{{S}_{0}},$$

where ${S}_{0}$ is the signal without diffusion encoding and $S(b)$ is the signal acquired with diffusion weighting characterized by the $b$*-*value, which quantifies the strength of diffusion weighting. The $b$*-*value depends on the gradient strength $G$, pulse duration $\delta$ and separation of the diffusion-encoding gradients $\Delta$:

2 $$b={\gamma }^{2}{G}^{2}{\delta }^{2}\left(\Delta -\frac{\delta }{3}\right),$$

In the following, we focus on the signal components corresponding to diffusion perpendicular and parallel to the foil layers, which reflect the restricted and free diffusion directions in the phantom, respectively.

### Perpendicular component (finite-pulse PGSE case)

To describe the signal attenuation arising from diffusion perpendicular to the confining planes, we consider the standard pulsed‐gradient spin‐echo (PGSE) sequence with two rectangular gradient pulses of duration $\delta$ separated by diffusion time $\Delta$. The time-integrated gradient waveform,

3 $$k(t)=\underset{0}{\overset{t}{\int }}g(t{\prime})dt{\prime},$$

takes the piecewise form:

4 $$k(t) = \left\{ {\begin{array}{*{20}l} {gt,} \hfill & {0 \le t \le \delta ,} \hfill \\ {g\delta ,} \hfill & {\delta < t \le \Delta ,} \hfill \\ {g\delta - g(t - \Delta ),} \hfill & {\Delta < t \le \Delta + \delta ,} \hfill \\ {0,} \hfill & {{\mathrm{otherwise}}{.}} \hfill \\ \end{array} } \right.$$

**Fig. 1:**
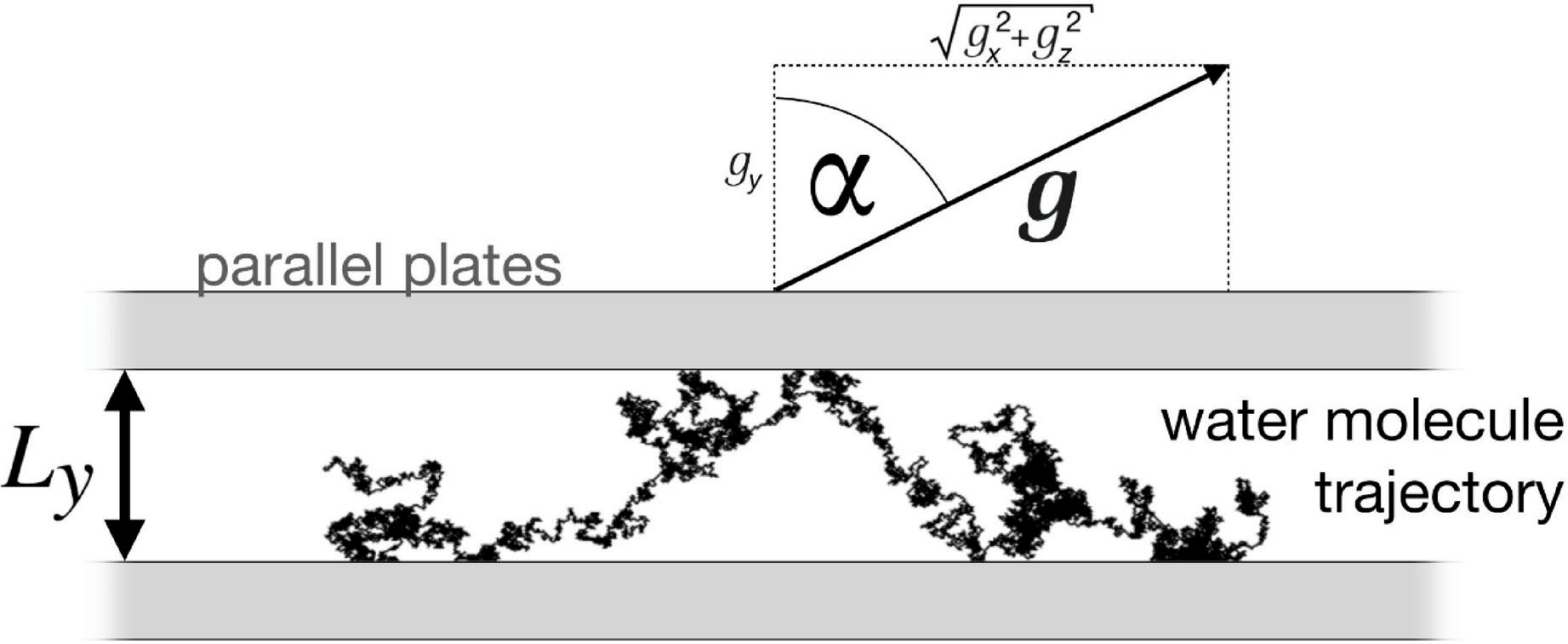
Schematic representation of restricted diffusion in a laminar system with an example trajectory of water molecule diffusing between two infinite parallel plates separated by a thin water layer of thickness ${L}_{y}$. The angle $\alpha$ represents the orientation of the diffusion gradient field relative to the plates.

When the gradient is applied at an angle $\alpha$ relative to the plane surfaces (see Fig. 1), only the normal component ${g}_{\perp }=G\mathrm{cos}\alpha$ probes the restricted direction, where $G$ denotes the diffusion gradient amplitude. For diffusion between two parallel, perfectly reflecting plates separated by distance $L$, the perpendicular signal attenuation can be written as a modal expansion over the eigenmodes of the 1D Laplacian. Following the multiple-correlation-function formalism of Grebenkov (Eqs. (48), (51) and (65) in^20^), this expansion directly leads to the double-integral representation of the second moment:

5 $$ln {E}_{\perp }(\alpha )=-{(\gamma {g}_{\perp })}^{2}\sum_{m=\mathrm{1,3},5,...}^{\infty }{W}_{m}\underset{0}{\overset{T}{\int }}\underset{0}{\overset{T}{\int }}k(t) k(t{\prime}){ \mathrm{e}}^{-{\lambda }_{m}\left|t-t{\prime}\right|} dt dt{\prime}$$

where $T=\Delta +\delta$ and the geometric weights are

6 $${W}_{m}=\frac{8}{{\pi }^{2}{m}^{2}} ,$$

and

7 $${\lambda }_{m}=\frac{{\pi }^{2}{m}^{2}D}{{L}^{2}}$$

are the characteristic relaxation rates of the perpendicular diffusion modes and $D$ denotes a diffusion coefficient.

In this work, Eq. (5) is evaluated numerically using the Multiple Correlation Function Approach Library (MCFAL)^20,21^, available at: https://pmc.polytechnique.fr/pagesperso/dg/MCF/MCF_e.htm.

The double time integral in Eq. (5) explicitly accounts for the finite duration of the gradient pulses. This expression reduces exactly to the Tanner–Stejskal^22^ restricted diffusion serie in the narrow‐pulse limit $\delta <<\Delta$. However, in the present experiments $\delta$ is not negligible ($\delta =4.5 ms$, $\Delta =16\text{ ms}$), placing the system in the finite‐pulse regime, where the classical narrow‐pulse expression is not accurate.

### Combination with the parallel (unrestricted) component

Motion parallel to the plates is unrestricted and contributes the standard Gaussian attenuation:

8 $${E}_{\parallel }(\alpha )=\mathrm{exp}\left[-(\gamma G \mathrm{sin}\alpha {\delta }^{2}\left(\Delta -\frac{\delta }{3}\right) D)\right],$$

which is the parallel part of Eq. (5) of Tanner and Stejskal^22^, including the finite‐pulse correction $\left(\Delta -\delta /3\right)$.

The total signal attenuation in the laminar system is therefore:

9 $$E\left( \alpha \right) = E_{\parallel } \left( \alpha \right) E_{ \bot } \left( \alpha \right),$$

which smoothly spans the entire restricted diffusion regime.

## Materials and methods

### Stacked-foil laminar phantom

**Fig. 2:**
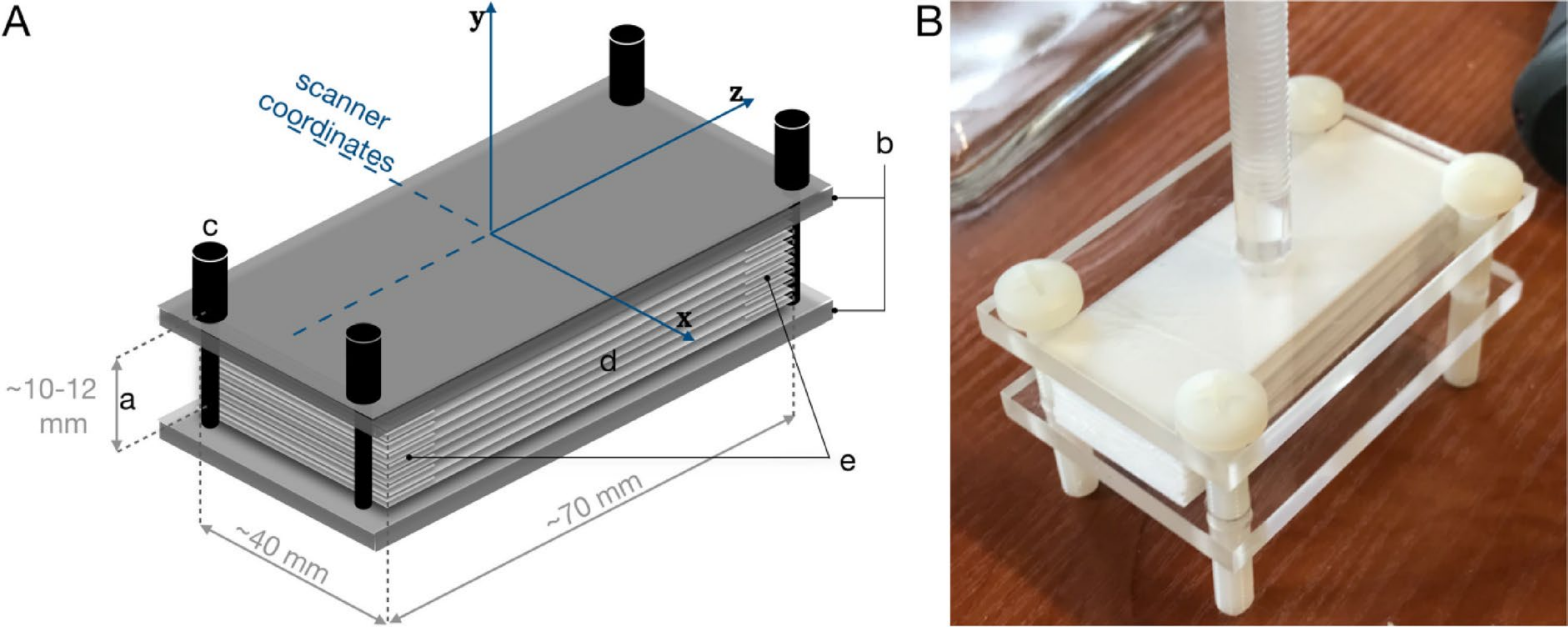
A schematic diagram (**A**) and real photo (**B**) of the phantom for studying planar diffusion and its orientation in the scanner. The stack of polyethylene foils (**a**) is pressed by two plastic plates (**b**). The distance between them is regulated by screws (**c**). The diagram shows an area with 600 (**d**) and 1800 layers of foil (**e**). The phantom is immersed in distilled water.

The idea behind the phantom construction is to create layers of water of controlled thickness between polyethylene foils. In this configuration, diffusion is primarily restricted perpendicular to the planes of the foils, while diffusion along the plane remains largely unrestricted, effectively generating a planar diffusion environment. The control of the water layer thickness is done by changing the distance between the plates. By knowing the thickness of a single foil, their number, and the height of the stack, the water layer thickness can be calculated, allowing the study of diffusion anisotropy as a function of the water layer thickness. Our phantom contains areas of different number of foil layers, resulting in various water layer thicknesses, which is schematically presented in Fig. 2. The thickness of a single foil layer was measured using an optical microscope by observing the foil immersed in paraffin.

The foils used for phantom construction were obtained by cutting commercial high-density polyethylene (HDPE) bags (Stella, Poland). HDPE is a linear polymer of ethylene (–CH₂–CH₂–) with a low degree of branching, which results in high crystallinity and mechanical rigidity. The material is chemically inert, hydrophobic, and impermeable to liquid water under experimental conditions. These properties make it suitable for creating well-defined water compartments without exchange across layers. The optical transparency of the foils also facilitates thickness determination using light microscopy.

The phantom was initially assembled empty, then filled with distilled water and subsequently degassed using a vacuum pump and ultrasound to remove any air trapped between the foils. The degassing process was repeated before each scanning session.

### MRI scanning protocol

Imaging was conducted at the Small Animal Magnetic Resonance Imaging Laboratory (Mossakowski Medical Research Institute, Polish Academy of Sciences, Warsaw, Poland) using a 7 T MRI scanner (*BioSpec 70/30 USR, Bruker Biospin GmbH*, Ettlingen, Germany). Three imaging sessions were performed, referred herein as experiments No.1, No.2, and No.3 respectively, each corresponding to a different foil stack compression (12.09 ± 0.03, 11.10 ± 0.02, and 10.11 ± 0.06 mm—Fig. 2A). During each session, the phantom was carefully positioned inside a circularly polarized, RF coil (inner diameter: 154 mm, model T11732V3, *Bruker Biospin GmbH*, Ettlingen, Germany), ensuring proper alignment to prevent tilting with respect to the horizontal plane. Prior to each session, wobble adjustments were performed on both coil channels to ensure optimal impedance matching and tuning.

The imaging protocol included a localizer scan (TriPilot), a T2-weighted acquisition (TurboRARE), and diffusion tensor imaging (DTI) using a spin-echo-based sequence (SE-DTI). The TurboRARE sequence was configured with the following parameters: repetition time (TR) = 3100 ms, echo time (TE) = 15 ms, RARE factor = 4, effective echo time (TE) = 30 ms, matrix size = 256 × 256, number of slices = 36, slice thickness = 2 mm, and spatial resolution = 0.3125 × 0.3125 mm. For the DTI acquisition, a spin-echo sequence was employed with the following parameters: TR = 5000 ms, TE = 27 ms, matrix size = 128 × 128, number of slices = 8, slice thickness = 2 mm, and spatial resolution = 0.9375 × 0.9375 mm. A total of 35 diffusion-weighted images were acquired, including five non-diffusion-weighted baseline images ($b_{0}$ images) and 30 diffusion-weighted images with diffusion gradients applied. The diffusion gradient field directions (${\boldsymbol{g}}$) were uniformly distributed over a sphere. Gradient parameters were set as follows: gradient pulse duration ($\delta$) = 4.5 ms, diffusion time ($\Delta$) = 16 ms, with a nominal $b$-value of 1000 s/mm^2^. Post-scan analysis of raw data indicated effective b-values ranging from 999 to 1022 s/mm^2^ for diffusion-weighted images and approximately 0.74 s/mm^2^ for baseline images. All measurements were conducted at a room temperature of 20.0 °C (± 0.5 °C).

### Diffusion tensor calculation

Diffusion images were converted from raw Bruker ParaVision to NifTi using an open source *dcm2niix* software^23^. Tensor calculations on a voxel-by-voxel basis were performed using the FSL software library^24^. Consequently, eigenvectors (${{\boldsymbol{e}}}_{1}$, ${{\boldsymbol{e}}}_{2}$ and ${{\boldsymbol{e}}}_{3}$) and eigenvalues ${(\uplambda }_{1}\ge {\lambda }_{2}{\ge \lambda }_{3})$ were obtained, along with parametrical maps, i.e. fractional anisotropy (FA), mean diffusivity (MD) and planary index (${C}_{p}$, as defined in^25^). For all other calculations, a Wolfram Mathematica software was used, with NIfTI images were imported via the QMRITools toolbox developed for the Wolfram language^26^.

### Detecting voxels with disrupted foil alignment

**Fig. 3:**
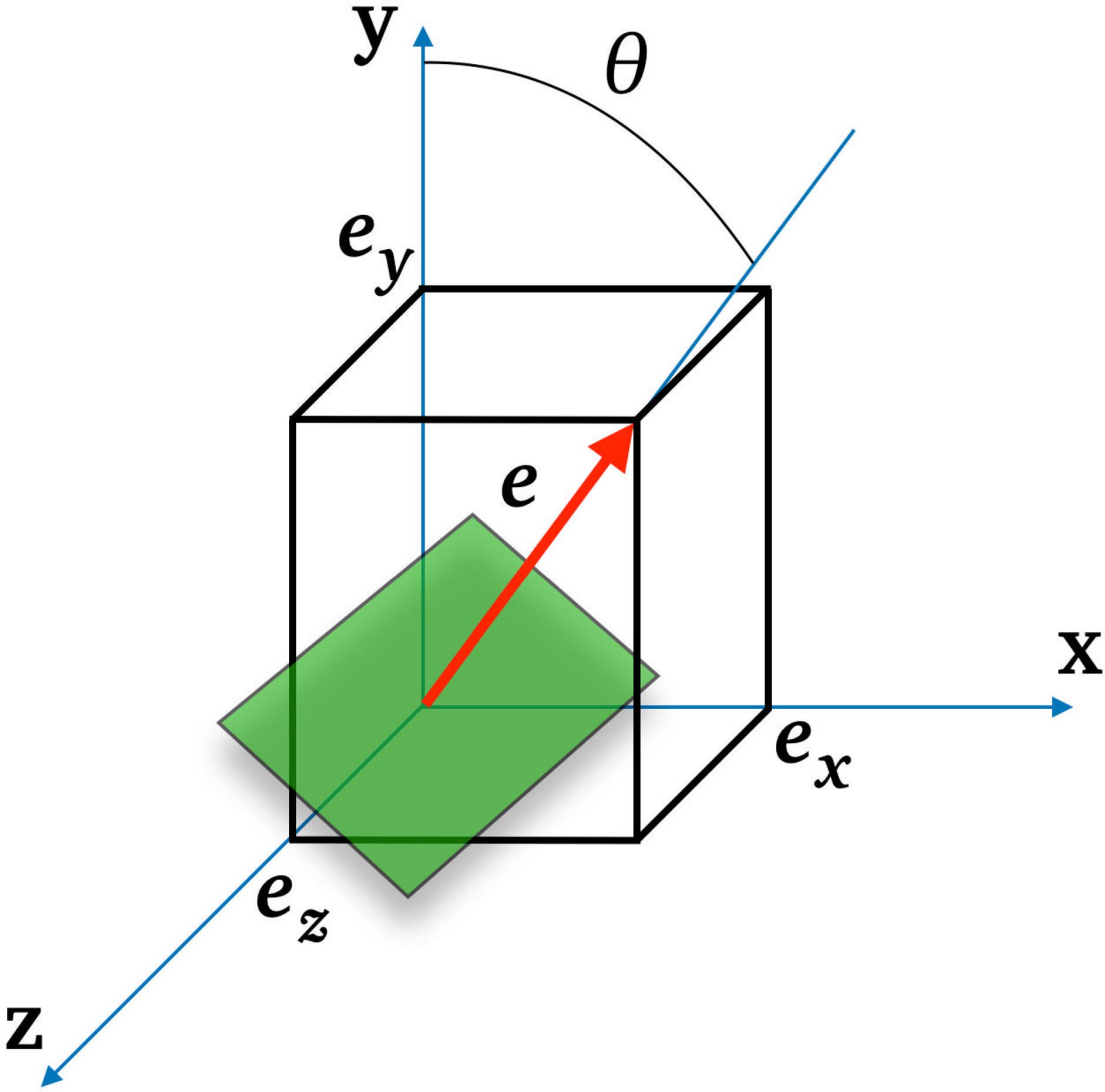
Schematic representation of the tilt angle $\theta$ of the vector ${\boldsymbol{e}}$, normal to the foil plane (highlighted in green), calculated from DWI data in the single voxel.

In a laminar system, the eigenvectors ${{\boldsymbol{e}}}_{1}$ and ${{\boldsymbol{e}}}_{2}$ define the water layer plane, while ${{\boldsymbol{e}}}_{3}$ is perpendicular to it (see Fig. 3), with ${\uplambda }_{1}$ and ${\uplambda }_{2}$ nearly equal and greater than ${\uplambda }_{3}$. Ideally, if the phantom is correctly positioned with laminar planes parallel to the plastic plates, ${{\boldsymbol{e}}}_{3}$ aligns with the scanner’s y-axis. For brevity, we denote the eigenvector ${{\boldsymbol{e}}}_{3}$ as ${\boldsymbol{e}}$, where ${\boldsymbol{e}}=\left({e}_{x},{e}_{y},{e}_{z}\right)$. Perfect alignment corresponds to $e=\left(\mathrm{0,1},0\right)$, so any nonzero ${e}_{x}$ or ${e}_{z}$ indicates misalignment. This misalignment, illustrated in Fig. 3, is quantified by the tilting angle $\theta$, given by ${\mathrm{cos}\theta =e}_{y}$. The angle $\theta$  is subsequently used to identify and exclude regions affected by local geometric distortions, forming the basis for the masking procedure described in next paragraph.

### Data processing

To define the region of interest (ROI) in the form of a mask, the phantom’s working area—i.e., the region containing the polyethylene foils—was manually delineated. This was done separately for areas with lower and higher foil density, while excluding regions with visible air bubbles. The mask was generated using the b₀ and T2-weighted morphological images, where air bubbles could be clearly identified and excluded. Within this delineated area, only voxels with an orientation angle (θ) below an arbitrarily chosen threshold of 1° were retained. This approach allowed for the identification of representative regions of the foil, free from unwanted geometric distortions.

### Inhomogeneities in water layers

Polyethylene foils are neither perfectly smooth nor rigid, which leads to variations in their thickness and the water layers between them. Surface irregularities from manufacturing, combined with bending, wrinkling, and warping when stacked, prevent uniform separation. As a result, water layers vary in thickness, creating thin regions where the foils nearly touch and thicker pockets where they are separated.

To account for submillimeter-scale water pockets between foils, diffusion within a 3D rectangular pocket with fully reflecting boundaries was modeled. The propagator $p\left({\boldsymbol{r}},t,{{\boldsymbol{r}}}_{0}\right)$ gives the probability density of a water molecule moving from an initial position ${{\boldsymbol{r}}}_{0}$ to a final position ${\boldsymbol{r}}$ over time $t$, while satisfying boundary conditions via the method of images. The resulting spin-echo attenuation $E$ was expressed in terms of elliptic theta functions. A full step-by-step derivation, including all assumptions and approximations, is provided in Appendix A.

**Fig. 4:**
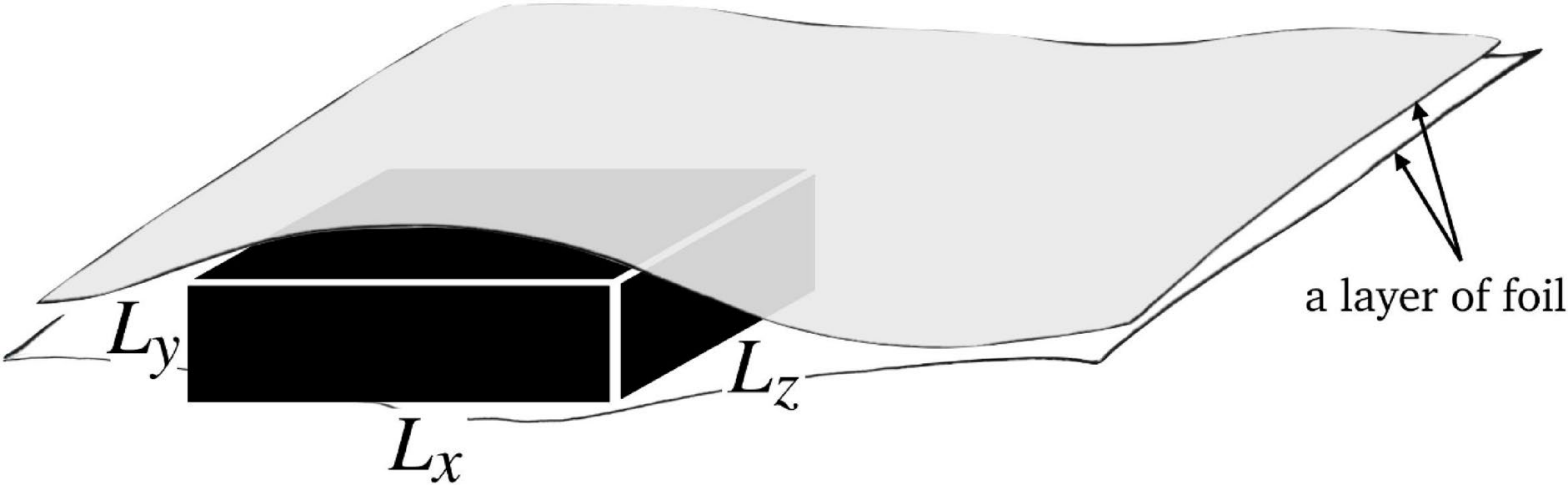
Schematic representation of a water pocket formed between two polyethylene foil layers. Due to surface irregularities and mechanical deformations (e.g., wrinkling or bending), the foils do not lie perfectly flat, leading to local variations in water layer thickness. In the simplest approximation, such a pocket is modeled as a rectangular prism with base ${L}_{x}={L}_{z}$ and height ${L}_{y}$.

The average size of water pockets can be estimated under different foil compressions. In the simplest case, a pocket is approximated as a rectangular prism with ${L}_{x}={L}_{z}$, while its height represents the estimated average water layer thickness ${L}_{y}$ (see Fig. 4). Based on the literature^22^, the signal attenuation $E$ for such rectangular pockets can be expressed as

10 $$E={\rho }_{x}{\rho }_{y}{\rho }_{z},$$

where:

11 $${\rho }_{s}=\frac{2-2\mathrm{cos}{a}_{s}}{{a}_{s}^{2}}+4{a}_{s}^{2}{\sum }_{n=1}^{\infty }{q}_{s}^{{\uppi }^{2}{n}^{2}}\frac{{1-\left(-1\right)}^{n}\mathrm{cos}{a}_{s}}{{\left({a}_{s}^{2}-{\uppi }^{2}{n}^{2}\right)}^{2}},$$

${a}_{s}=\gamma {G}_{s}\delta {L}_{s}$ and ${q}_{s}=\mathrm{exp}\left(-Dt/{L}_{s}^{2}\right)$ for $s=x,y,z$. Here, the time parameter $t$ corresponds to the PGSE diffusion time $\Delta$, i.e. the interval during which molecular displacement occurs between diffusion gradient pulses. The full derivation of the formula for signal attenuation $E$ in a three-dimensional rectangular well can be found in Appendix A. The derivation presented in Appendix A employs the method of images to satisfy the boundary conditions in a three-dimensional rectangular well, in a manner analogous to the image method previously applied by Özarslan et al.^27^.

## Results

**Fig. 5:**
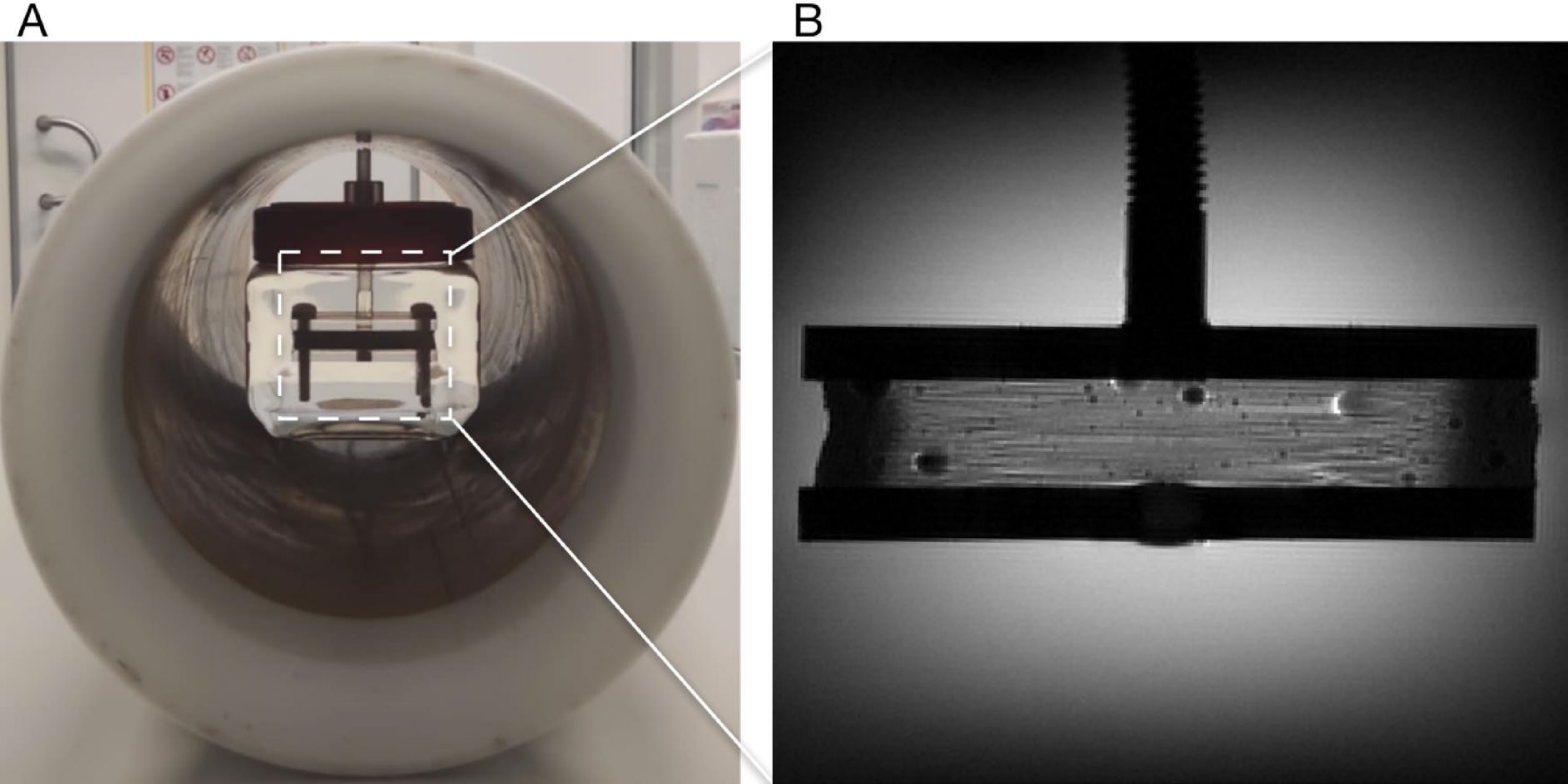
Phantom placed inside the MRI coil (**A**) and the corresponding T2-weighted image in the yz projection (**B**).

To illustrate the setup, actual images of the phantom are presented in Fig. 5.

### Assessment of foil orientation spatial distribution

The mean rotation angles $\langle {e}_{x}\rangle$ and $\langle {e}_{z}\rangle$ were calculated for each of the three experiments by averaging the values of all voxels within the phantom’s working area—specifically, the area containing the films—while excluding regions with visible air bubbles, as shown in Fig. 5D. The results, which quantify the misalignment of ${\boldsymbol{e}}$ from the ideal alignment with the scanner’s y-axis, are presented in Table 1.

**Table 1:** Mean values of $e_{x}$, $e_{z}$ and $\theta$ for experiments 1–3 at different foil compression levels.

	No.1	No.2	No.3	Average
$\left\langle {e_{x} } \right\rangle$	0.01 ± 0.02	0.01 ± 0.02	− 0.0 ± 0.05	0.01 ± 0.03
$\left\langle {e_{z} } \right\rangle$	− 0.01 ± 0.03	− 0.02 ± 0.03	− 0.01 ± 0.06	− 0.01 ± 0.04
$\left\langle \theta \right\rangle$	0.7° ± 0.2°	0.7° ± 0.2°	0.6° ± 0.2°	0.7° ± 0.2°

The average value of the angle $\theta$ represents the mean deviation of the foil surfaces relative to the scanner’s coordinate system.

### DW-MRI-derived parameters

**Fig. 6:**
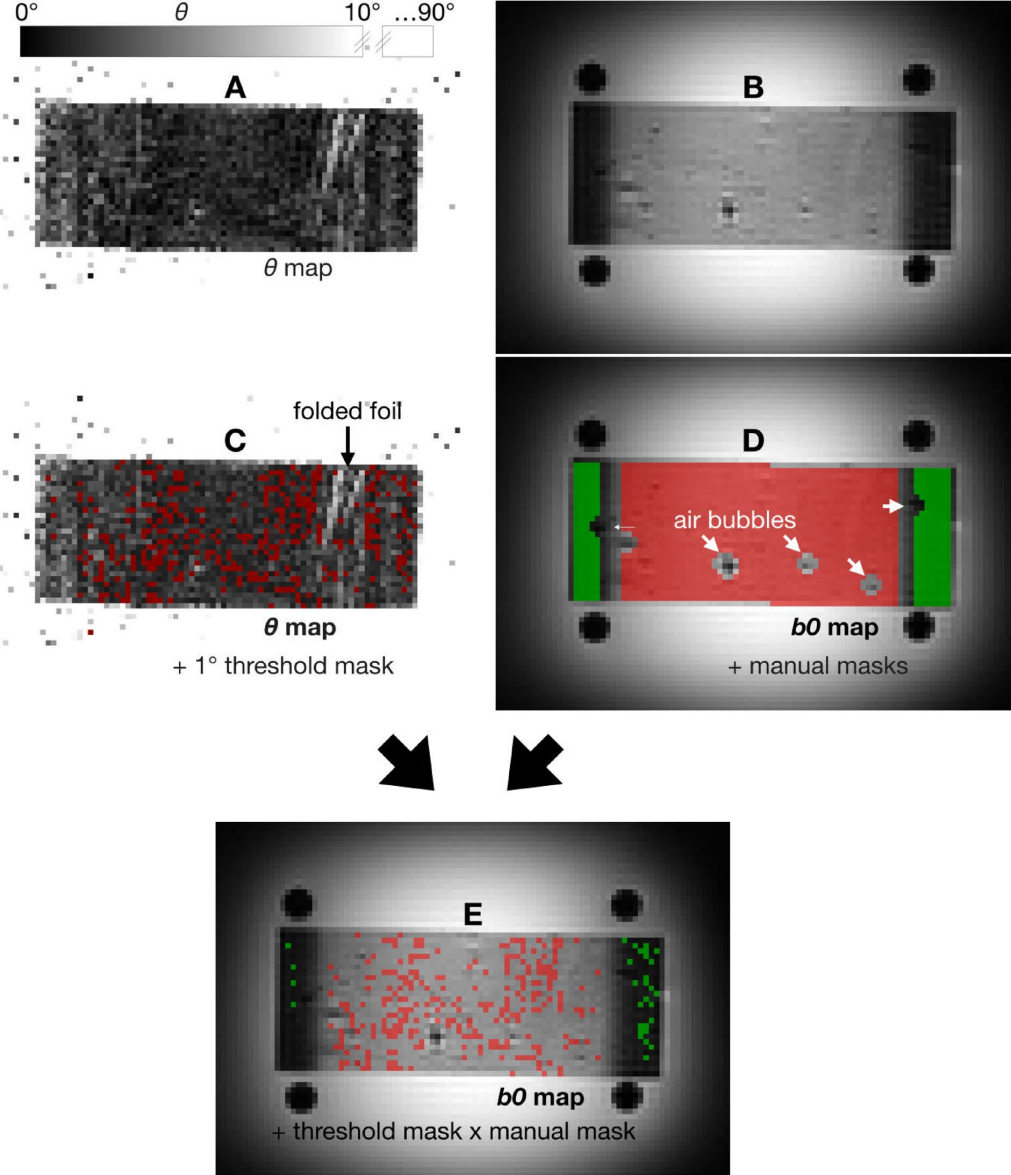
An example map of $\theta$ values for the phantom in the zx plane (**A**) with corresponding $b_{0}$ map (**B**). The region of interest was determined as the intersection of the mask created based on the $\theta$ <1° threshold (**C**) and the manually drawn mask, excluding areas with visible air bubbles (**D**). The red and green colors represent the mask for areas with lower and higher foil density, respectively (**D**, **E**).

To ensure that the analysis focused on representative regions of the phantom, a volume of interest (VOI) was defined by combining two masking approaches. Figure 6 illustrates masking process, showing an example map along with the corresponding image and the resulting masks.

**Fig. 7:**
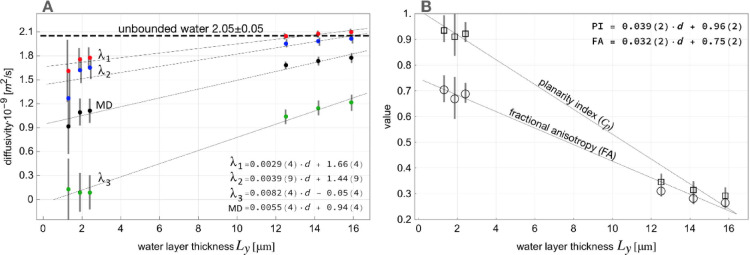
The results obtained on the phantom presenting diffusivity in the principal directions (**A**) where $\lambda_{3}$ is a value perpendicular to the foil surface, and the fractional anisotropy FA with the planarity index $C_{p}$ (**B**). Vertical gray bars indicate the standard deviation. The uncertainty of $L_{y}$ is ± 1.0 μm. For fitted linear parameters $p$-value >  > 0.05.

For quantitative analysis, values of the scalar maps FA, MD, $\lambda_{1}$, $\lambda_{2}$, $\lambda_{3}$ and $p_{i}$ were measured in the selected VOIs. The obtained results are presented in Fig. 7.

### Estimation of water layer thickness

The thickness of a single polyethylene foil layer was estimated using a DM 1000 LED microscope (*Leica Camera AG*, Germany), and a detailed analysis of the measurement process is presented in Appendix B. The estimated foil thickness was 4.3 µm, with a standard deviation of ± 1.0 µm, based on the spread of the measured values. Knowing the geometric dimensions of the phantom and applying error propagation calculations, the water layer thickness was estimated in the region with higher foil density as 2.4 µm, 1.9 µm, 1.3 µm, and in the central region with lower foil density as 15.9 µm, 14.2 µm, 12.5 µm, respectively, with an uncertainty of ± 1.0 µm, for experiments No. 1, No.2 and No.3.

### Effective diffusion gradient amplitude validation

In diffusion MRI, the actual b-value may deviate from the nominal value prescribed by the scanner due to spatial gradient nonlinearities and cross-terms between diffusion and imaging gradients^28–30^. These effects are inherent to all gradient systems and arise from engineering constraints and gradient coil design. As a result, diffusion-sensitizing gradients may vary in magnitude and orientation across the field of view. Neglecting these deviations can introduce systematic biases in estimated diffusion metrics such as mean diffusivity (MD) and fractional anisotropy (FA). To mitigate these effects, we apply a simplified empirical calibration based on free water measurements to obtain effective diffusion-weighting parameters ensuring internal consistency of the model fitting. Using this assumption, the free-water signal attenuation curve was fitted with the mono-exponential diffusion model, yielding an effective gradient amplitude of $G$ = 214.4 ± 6.6 mT/m, compared to the nominal value of $G$ = 237.42 mT/m. This corresponds to a relative deviation of approximately 10%.

From this fitted value, the effective $b$-value was computed to be 966 ± 60 s/mm^2^. This result is in good agreement with the value found in the scanner metadata, 1010 ± 8 s/mm^2^, which was used by the FSL *dtifit* tool for diffusion tensor reconstruction.

The effective gradient amplitude obtained from this fitting was used in all subsequent analyses.

### Measuring the accuracy of proposed diffusion model

**Fig. 8:**
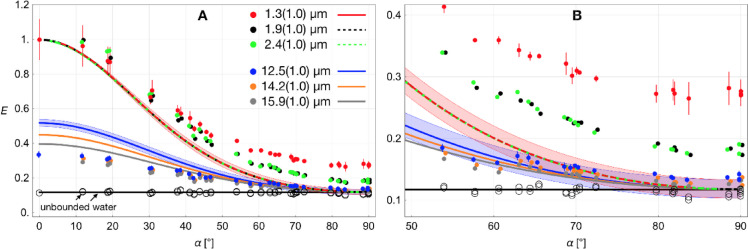
The results obtained on the phantom presenting the diffusion signal attenuation $E$ in a function of angle $\alpha$ between diffusion gradient field direction and the surfaces of the foils for six different water layer thicknesses (dots) and the theoretical prediction from Eq. (9) (dashed and solid lines). The uncertainty of the measurement, resulting from the repeated experiment, is shown using vertical lines for each point. The uncertainty of the theoretical fit is represented as a red and blue shadow around the red and blue lines and is at the level of the thickness of the drawn lines for all curves. To maintain clarity, only representative uncertainties are displayed.

In order to evaluate the agreement between experimental data and the model described by Eq. (9), the signal attenuation $E\left( \alpha \right)$ was calculated voxel-wise as the ratio $S\left( b \right)$/$S_{0}$ where each diffusion-weighted signal was normalized by its corresponding non-diffusion-weighted image. The resulting attenuation values were subsequently averaged within the selected regions of interest as a function of the angle $\alpha$. The theoretical fits to the experimental data are shown in Fig. 8. The value of the dimensionless restricted-diffusion parameter $p$ differed across the samples due to different layer spacings, placing the smallest-spacing samples in the restricted regime ($p > > 1$) and the largest spacings near the free-diffusion regime ($p < < 1$). The theoretical curves in Fig. 8 therefore represent the physically correct model across all diffusion regimes. This confirms that samples with small $L$ are firmly in the restricted regime, while samples with large $L$ approach the regime of free diffusion (see Table 2).

**Table 2:** Values of the dimensionless parameter $p$ and the corresponding diffusion regime for layer spacing $L$.

$\sim L \left[ {{\mu m}} \right]$	$\sim p = D \Delta /L^{2}$	Regime
1.3 1.9 2.4 12.5 14.2 15.9	19.2 8.9 5.6 0.21 0.16 0.13	strongly restricted diffusion restricted transition–restricted transition → nearly free nearly free nearly free

The uncertainty of the $E$ for experimental points was measured as the difference between the results obtained from a repeated experiment for the strongest foil stack compressions. The second experiment was repeated after approximately 17 months with the same phantom. The uncertainty of the theoretical prediction from formula (9) was determined through error propagation due to the uncertainties in the diffusion coefficient, water layer thickness and $G$. The uncertainty in the water layer thickness depends on the degree of compression of the foil within the phantom.

### Inhomogeneities in water layers

**Fig. 9:**
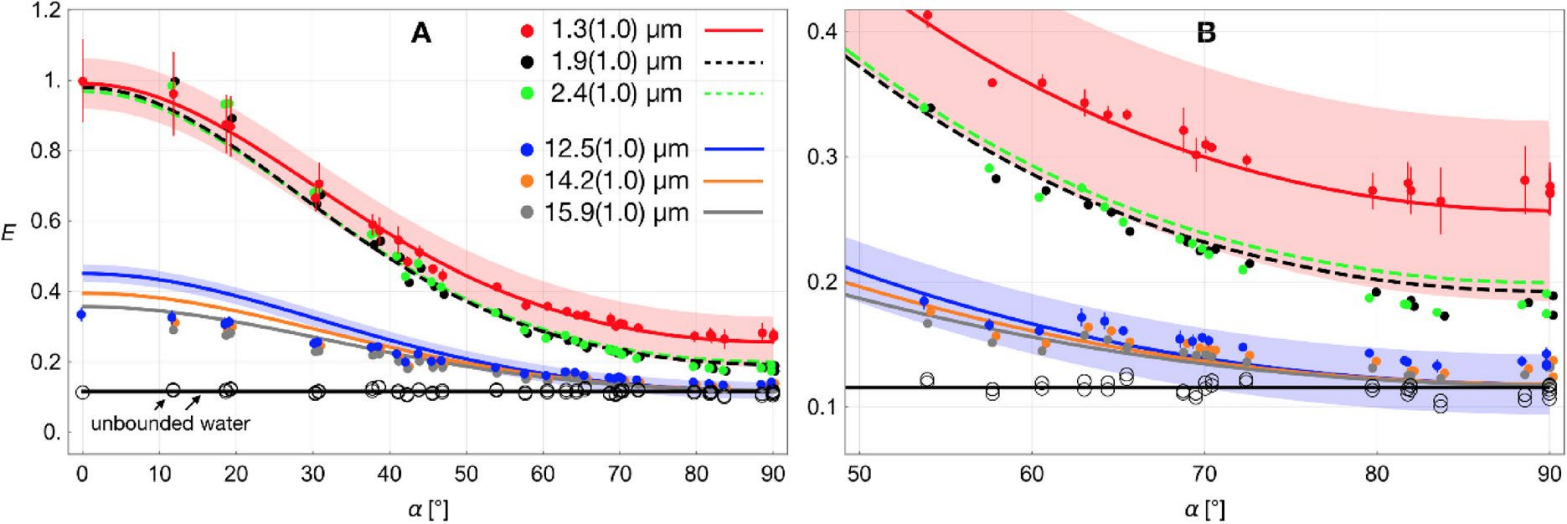
The fitting result to the measurement points based on the theory from Eq. 11 (**A**) and a magnified view for small $\alpha$, corresponding to the gradient nearly parallel to the foil surfaces (**B**). The measurement uncertainty, calculated from repeated experiments, is represented by vertical lines at each point. The shaded red area indicates an example lowest uncertainty in the fitted lines due to the uncertainties in the determined $L_{x} , D$, $G$ and $L_{y}$ values.

**Fig. 10:**
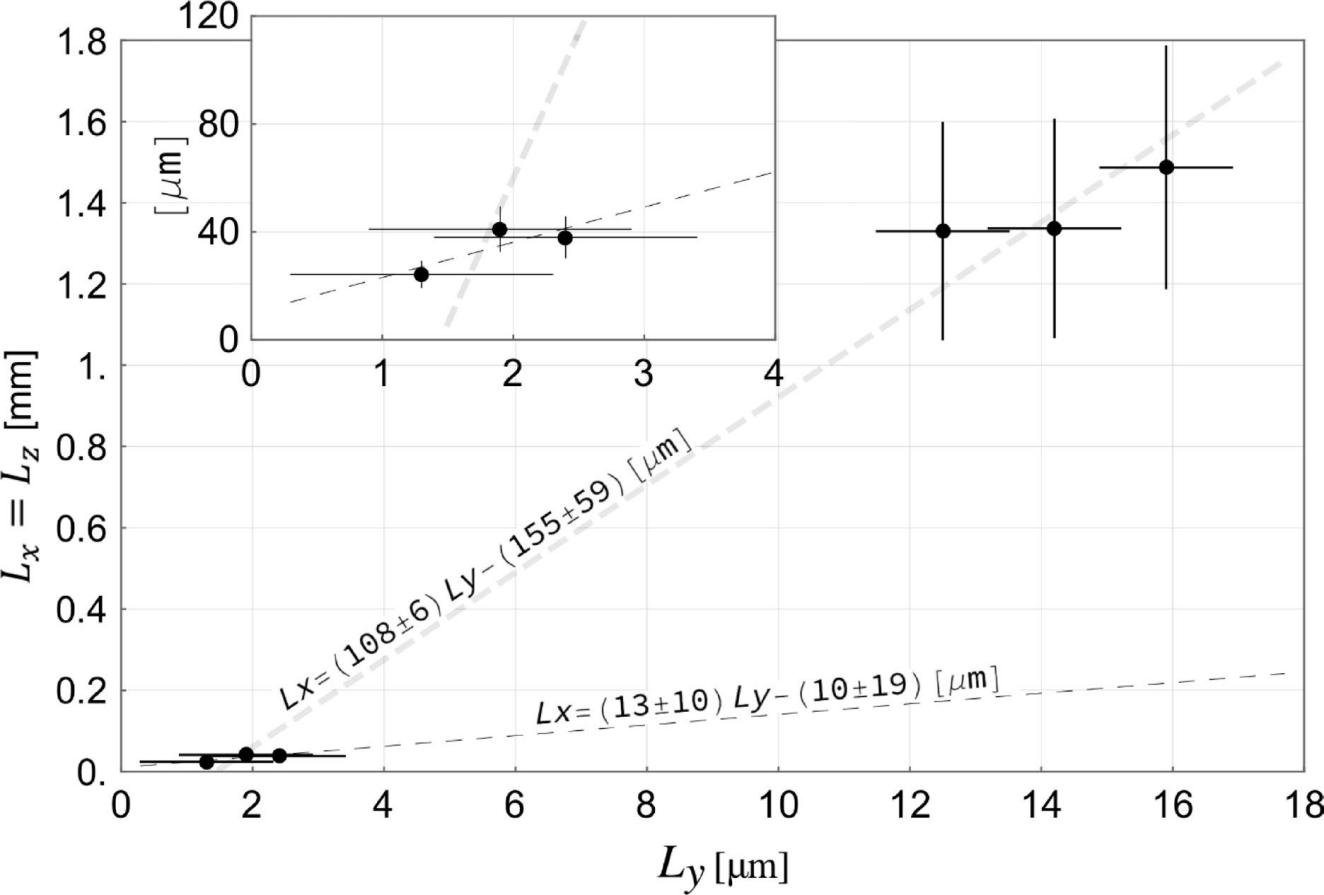
Determined sizes of the observed inhomogeneities, identified as water pockets of size $L_{x}$ as a function of the water layer thickness $L_{y}$. The inset shows a magnified view for small values of $L_{y}$. The light gray dashed line represents the fit to all points, whereas the dark gray dashed line corresponds to a linear fit restricted to small water pockets with reliable $L_{x}$ estimates.

Relation (10), with $L_{y}$, was fitted to the experimental data using least squares. Due to the summation term in Eq. (10), the fit was performed numerically. Since $L_{x} = L_{z}$ was assumed earlier, we now denote both simply as $L_{x}$. This parameter was estimated by minimizing the sum of squared differences between measured and theoretical values (Fig. 9). Uncertainty was defined as the range of $L_{x}$ for which the error increased by 1 from its minimum. The fitted average water pocket sizes $L_{x}$ for various sample thicknesses are shown in Fig. 10.

A clear positive correlation between $L_{x}$ and $L_{y}$ was observed, indicating that the lateral size of the water inhomogeneities increases with the spacing between the foil layers. This behavior is consistent with expectations: the more the foils are compressed (i.e., the smaller the $L_{y}$), the smaller the resulting pockets of water. For the largest pockets, $L_{x}$ exceeds the mean squared displacement for the given diffusion time, resulting in high uncertainty. Therefore, an additional linear fit was performed including only smaller pockets ($L_{x}$ ≲ 50 μm), where $L_{x}$ can be reliably estimated.

## Discussion and summary

Water diffusion between the laminar layers is directionally restricted, and the extent to which this restriction is expressed in the diffusion-weighted signal is governed by the dimensionless parameter $p = D\Delta$/$L^{2}$. In our experiments, the samples span both the restricted ($p > > 1$) and near-free ($p < < 1$) regimes. Because the gradient pulses are not short compared to $\Delta$, the classical narrow-pulse Tanner–Stejskal expression (Eq. (5) in^22^) is not applicable. We therefore model the perpendicular component of the signal using the finite-pulse restricted-diffusion formulation, while the parallel component follows the standard Gaussian attenuation. This combined model accurately reproduces the measured angular signal attenuation and enables quantitative estimation of the layer spacing $L$.

### Diffusion anisotropy and phantom validity

The significant reduction of the third eigenvalue ($\lambda_{3}$) compared to $\lambda_{1}$ and $\lambda_{2}$ is consistent with directional constraints parallel to the polyethylene HDPE foil surfaces, supporting the phantom project validity in simulating laminar diffusion^2,3^. In addition to FA, the planarity index $C_{p}$ further characterized the anisotropy pattern of the phantom. For the thinnest water layers, $C_{p}$ reached values close to 1, indicating strongly planar diffusion restricted to the foil surface. In regions with lower foil density, $C_{p}$ decreased to ~ 0.3. This reduction suggests that diffusion in these areas was less strictly confined to two dimensions, likely due to increased inter-layer spacing and formation of water pockets, which facilitate partial isotropic or linear diffusion components. Hence, PI provided complementary evidence that microstructural irregularities in the phantom reduce the degree of laminar restriction. Our results confirm the presence of strong diffusion anisotropy in layered structures and demonstrate that key features observed in biological laminar systems can be replicated. Notably, the observed deviation from the Tanner–Stejskal prediction reflects phenomena similar to those described by Ye et al.^1^, where anomalous diffusion arises due to microscale inhomogeneities, consistent with recent findings that classical diffusion models fail to fully capture compartmental complexity^31,32^.

### Structural heterogeneities and signal divergence

Both Figs. 8 and 9 reveal that the fitting quality remains limited, especially for perpendicular gradients, where deviations clearly exceed the noise level. Despite voxel-wise normalization improved the overall consistency of the data and eliminated non-physical baseline offsets, the systematic differences between the model and the measurements remain. This indicates that—even with the extended model—additional physical or experimental factors not included in the formulation influence the measured signal. Accordingly, the fitted parameters should be interpreted with this limitation in mind.

The contribution of trapped air bubbles, although minimized through degassing, remains a minor source of uncertainty. We propose that the principal cause of signal divergence lies in local heterogeneities, potentially water pockets or uneven compression between foils—an effect reminiscent of findings in layered lipid bilayers^6^. Additionally Infrared spectroscopy, thermogravimetric analysis (TGA), and scanning electron microscope (SEM) analyses excluded the presence of bound water in the foil material, eliminating a significant confounding factor (see Appendix C).

These observations align with recent concerns raised by Rockland and Shamir et al.^3,4^, who emphasized the complexity of modelling diffusion in laminar brain structures. The assumption of idealized geometry proves to be insufficient, as even subtle structural variations can measurably affect diffusion signal. By replicating such laminar irregularities, the phantom provides a controlled system for validating analytical models of restricted diffusion, which may help refine diffusion models used in layered biological tissues. The robustness of the phantom design was further addressed by geometric analyses of the foil stack. Average misalignments of the layers, derived from $\theta$ angle maps, did not exceed 1.1° in any direction, with standard deviations suggesting that foil waviness had a greater impact than positioning errors. The careful selection of regions of interest, combining threshold-based and manual masking, ensured that diffusion parameters were extracted from representative, well-aligned zones.

The interaction of HDPE with water is limited to physical confinement, as the material is hydrophobic and chemically inert. Minor structural irregularities, however, can promote the formation of microscopic water pockets, which we observed in our data. Compared to alternative materials such as glass, polytetrafluoroethylene or polycarbonate, HDPE foils offer the advantages of flexibility, optical transparency for thickness assessment, low cost, and impermeability to water, making them particularly suitable for constructing laminar phantoms.

### Estimation of effective diffusion gradient parameters

The estimation of effective diffusion gradient parameters was performed to ensure internal consistency between the experimental data and the signal model. In diffusion MRI, deviations between nominal and effective diffusion weighting may arise from modeling inaccuracies, gradient nonlinearities, and sequence-dependent gradient interactions. To partially account for these effects, we used free water as an internal reference and applied an empirical calibration of the diffusion weighting.

Using corrected b-values led to an improvement in fitting precision and more consistent interpretation of the phantom data across all gradient orientations. Although advanced diffusion MRI pipelines often apply voxel-wise gradient nonlinearity corrections^33^ resulting in spatially varying b-values and b-matrices, such a full spatial correction was not implemented here. Instead, a global calibration was used, reflecting the controlled phantom setup, the limited region of interest within the field of view, and the aim of achieving internally consistent model fitting rather than hardware-level gradient-field characterization. Consequently, the estimated effective gradient amplitude should be interpreted as an empirical correction parameter rather than a voxel-wise physical calibration.

### Model-based analysis of water pockets

Model-based analysis of diffusion inhomogeneities suggests the formation of water pockets between layers, with lateral dimensions ranging from 28 to 1500 μm depending on foil compression. A proportional relationship between $L_{x}$ and $L_{y}$ was found, supporting the idea that stronger compression (i.e., lower $L_{y}$) reduces the size of these pockets. For the largest pockets, L cannot be reliably estimated due to the limited diffusion time; an additional linear fit including only smaller pockets ($L_{x}$_x_ ≲ 50 μm) confirms the proportional relationship in regions where estimates are accurate. Similar to recent efforts in compensating for systematic distortions in diffusion MRI acquisition, such as the gradient nonlinearity correction proposed by Kanakaraj et al.^34^, our study highlights the importance of accounting for subtle structural or instrumental imperfections—here, in the form of phantom-intrinsic laminar irregularities—that can significantly affect derived diffusion metrics.

### Relation to previous models and literature

The extended laminar diffusion model proposed here builds upon a broader body of work investigating restricted diffusion in layered or periodically structured systems. Previous studies^20,35–39^ have developed analytical or numerical approaches to treat restricted or anisotropic geometries. These models provide important theoretical foundations but often rely on idealized assumptions such as perfectly homogeneous layers or strictly Gaussian propagators. In contrast, the present model explicitly accounts for the presence of structural irregularities (e.g., inter-foil water pockets) and enables angular analysis of the signal, which is essential in realistic phantom experiments. By situating the proposed framework within this continuum of prior work, it can be concluded that the present contribution provides a complementary step: from idealized laminar diffusion theory towards experimentally validated models that incorporate realistic microstructural complexity.

### Summary and outlook

In summary, while established corrections and numerical libraries for diffusion signal modeling remain useful in specific conditions, their limitations under the present experimental design highlight the relevance of the proposed model. The phantom thus offers a robust platform for studying laminar diffusion processes and for benchmarking advanced diffusion MRI methodologies.

## Limitations of the study

A limitation of the study was that measurements were performed with one b-value, which may affect the determination of the diffusion parameters. Additionally, the phantom structure, although designed to mimic laminar systems, may not capture the full complexity of biological tissues, particularly in terms of dynamic fluid exchange and cellular heterogeneity. Residual air bubbles and local foil irregularities, despite masking efforts, may have introduced minor influence on results. Finally, the static nature of the phantom does not allow assessment of time-dependent or perfusion-related effects, which also could be relevant in in vivo studies.

## Conclusions

A novel, adjustable phantom was constructed from stacked polyethylene HDPE foils immersed in water, creating water layers with thicknesses ranging from ~ 1 to 16 μm. Diffusion-weighted MRI measurements demonstrated restricted diffusion perpendicular to the foil planes. The measured signal attenuation deviated from classical diffusion theory predictions, motivating the proposal of a modified model that incorporates submillimeter-scale water pockets between layers, with lateral dimensions ranging from 28 to 1500 μm depending on foil compression. This work provides a controlled experimental system for validating analytical models of restricted diffusion and may offer insights relevant to layered biological structures.

## Supplementary Information

Below is the link to the electronic supplementary material.

Supplementary Material 1.

## Data Availability

Data supporting the findings of this study are available from the corresponding author upon reasonable request.
