# Supplementary material for: A phantom mimicking layered biological microenvironments investigated with 7T diffusion weighted MRI
Source: Sci Rep. 2026 Apr 16;16:17721. doi: 10.1038/s41598-026-47120-6 (PMC13247201; doi:10.1038/s41598-026-47120-6)
Supplement: Supplementary file 1 — Supplementary Material 1. [file 41598_2026_47120_MOESM1_ESM.pdf]

Appendix A

SIGNAL ATTENUATION IN A THREE-DIMENSIONAL RECTANGULAR WELL

In this appendix, we derive the analytical expression used to describe signal attenuation from diffusion confined within a rectangular water pocket, corresponding to the model introduced in the main text. For consistency with the main manuscript, Eqs. (10) and (11) retain their numbering from the main text, while equations labeled with the suffix "a" are introduced specifically in this Appendix.

We consider confined diffusion in a three-dimensional rectangular well with fully reflecting walls located at 0 and L_x, 0 and L_y, 0 and L_z in Cartesian coordinates. The components of $\boldsymbol{r}$ vector are denoted as (x,y,z). Using the method of images, point sources are placed at $\boldsymbol{r}_{kmn} = (x_k, y_m, z_n)$, where

$$x_k = \frac{1}{2}\left(2k + 1 - (-1)^k\right)L_x + (-1)^k x_0, \quad k = 0, \pm 1, \pm 2 \dots . \tag{1a}$$

The expressions for y_m and z_n are defined analogously. The probability density for $0 < x < L_x$, $0 < x_0 < L_x$ (similarly for y, y_0, z and z_0) is

$$p(\boldsymbol{r}, t, \boldsymbol{r}_0) = \frac{1}{(4\pi D t)^{\frac{3}{2}}} \sum_{k=-\infty}^{\infty} \sum_{m=-\infty}^{\infty} \sum_{n=-\infty}^{\infty} \exp\left(-\frac{|\boldsymbol{r} - \boldsymbol{r}_{kmn}|^2}{4Dt}\right), \tag{2a}$$

where $p(\boldsymbol{r}, t, \boldsymbol{r}_0) = 0$ outside the well. This separability allows the three-dimensional propagator to be written as a product of independent one-dimensional contributions

$$p(\boldsymbol{r}, t, \boldsymbol{r}_0) = p_x p_y p_z \tag{3a}$$

with each component:

$$p_s = \frac{1}{2L_s}\left(\vartheta_3\left(\frac{\pi(s - s_0)}{(2L_s)}, q_s^{\pi^2}\right) + \vartheta_3\left(\frac{\pi(s + s_0)}{(2L_s)}, q_s^{\pi^2}\right)\right), \tag{4a}$$

where $s = x, y, z$,

$$q_s = \exp\left(-\frac{Dt}{L_s^2}\right), \tag{5a}$$

and ϑ_3 is the elliptic theta function defined as:

$$\vartheta_3(\beta, k) = 1 + 2\sum_{n=1}^{\infty} k^{n^2} \cos(2n\beta), \tag{6a}$$

In Eq. (1a), the dependence on the initial position r_0 is implicit, because the image sources r_{kmn} already represent all reflections of a point originally located at r_0. The step from Eq. (3a) to Eq. (4a) follows from rewriting the one-dimensional sums in terms of the Jacobi theta function, which in this context corresponds to the Fourier transform of the propagator, not the propagator itself.

The propagator obtained above is next used to compute the diffusion-induced spin-echo attenuation, which corresponds to the quantity measured experimentally. To calculate the attenuation factor E of the spin-echo amplitude due to the nuclear spin diffusion, we use the approximation from [31], defining

$$E = \frac{1}{V}\int_{V_0} \int_{V} p(\boldsymbol{r}, t, \boldsymbol{r}_0) \exp\left(-\mathrm{i}\gamma\delta \boldsymbol{G} \cdot (\boldsymbol{r} - \boldsymbol{r}_0)\right) \, \mathrm{d}V \mathrm{d}V_0. \tag{7a}$$

Supplementary materials

The Cartesian components can be factorized. Introducing the dimensionless parameters $a_s = \gamma G_s \delta L_s$ and changing the integration variables to dimensionless ones, the attenuation E becomes

$$E = \rho_x \rho_y \rho_y. \tag{10}$$

Here, the attenuation factor is expressed as a product of independent contributions along each Cartesian direction, and

$$\rho_s = \frac{1}{2}\int_0^1 \int_0^1 \exp\big(-\mathrm{i}a_s(u-u_0)\big)\left(\vartheta_3\left(\frac{\pi(u-u_0)}{2}, q_s^{\pi^2}\right) + \vartheta_3\left(\frac{\pi(u+u_0)}{2}, q_s^{\pi^2}\right)\right)\mathrm{d}u\mathrm{d}u_0. \tag{8a}$$

The explicit series representation of ρ_s is:

$$\rho_s = \frac{2-2\cos a_s}{a_s^2} + 4a_s^2 \sum_{n=1}^{\infty} q_s^{\pi^2 n^2} \frac{1-(-1)^n \cos a_s}{(a_s^2 - \pi^2 n^2)^2}. \tag{11}$$

Equation (12) provides the explicit form used in the numerical evaluation of the model presented in the main text.

Appendix B

MEASUREMENT OF THE POLYETHYLENE FOIL THICKNESS

The thickness of a single layer of polyethylene foil was estimated using a microscope *DM 1000 LED* (*Leica Camera AG*, Germany). To prepare the sample, a strip of polyethylene foil was placed on a heating table set to around 50°C, and then a layer of paper towel was placed on the foil, onto which melted paraffin was poured. Thanks to the heating table, the paraffin did not solidify immediately. The strip of foil, along with the paper towel in paraffin, was wound onto a paper stick. The entire setup was then placed in a silicone mold, and more paraffin was added. This resulted in a paraffin cube with a side length of 1 cm (Figure 11A), which was then cut into thin slices using an automatic knife for biological specimens. Each thin slice of the paraffin cube was briefly placed in warm water straighten it, as it had curled during cutting. The prepared specimens were then observed under an optical microscope (B).

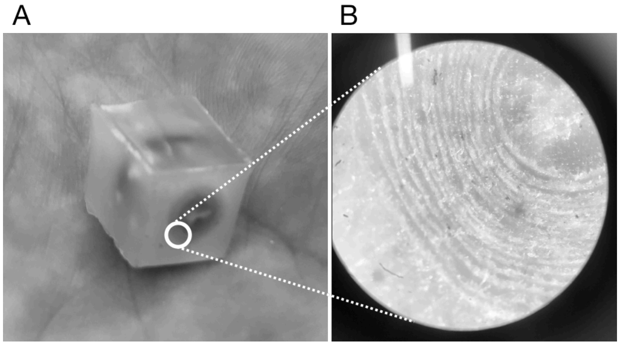

Figure 11. A paraffin cube with dimensions 1x1x1 cm³ containing rolled polyethylene foil inside (A) and a view at 10x magnification after cutting a thin slice from this cube (B).

To improve visibility, the sample was stained with hematoxylin (Figure 12A). Then, a fragment of the single foil was selected, which in visual assessment seemed the most representative, i.e., it was aligned along the cutting direction of the knife, with a thickness comparable to other areas, with no significant differences observed along its entire length, caused by possible "tearing" by the cutting knife. On this image, the edges of the foil were manually drawn as dashed lines (B). The white segments stretched between the dashed lines are markers of the foil's thickness at several points, used to construct the histogram. Further analysis was performed in the Wolfram Mathematica program, where, after loading the image, the pixel coordinates containing the dashed lines were identified. For the pixels containing the lower dashed line, a straight line was fitted using the least squares method, and then along the perpendicular to this line, the segments from each pixel of the lower dashed line were measured in steps of 10 pixels up to the upper dashed line. This was done by locating the intersection point of the line with the interpolated function to the pixels on the higher dashed line. In this way, a relatively uniform distribution of thickness measurement points was obtained along the entire visible length of the foil (see insert in Figure 12B). The distance calibration in the microscopic image was carried out using a micrometer slide with a micrometer scale. By measuring the length of the segments, the foil thickness d was estimated at 4.3 µm, with a standard deviation of 1.0 µm based on the spread of the measured values.

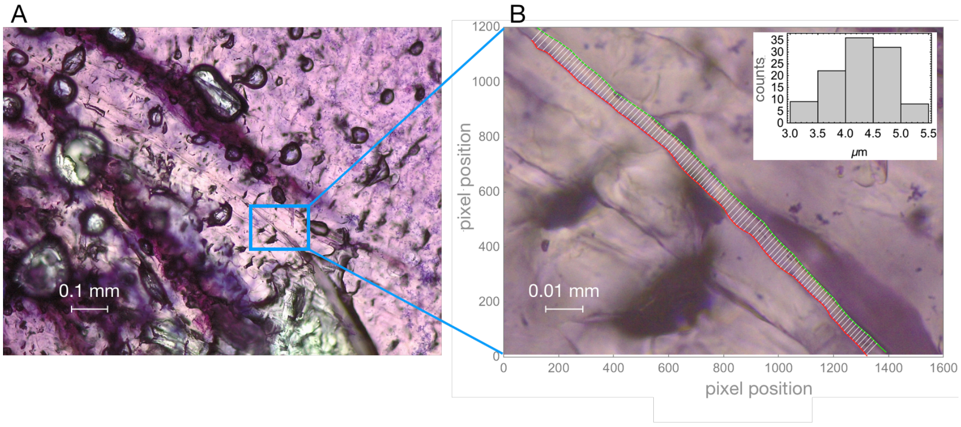

Figure 12. A paraffin cube with dimensions 1x1x1 cm³ containing rolled polyethylene foil inside (A) and a view at 10x magnification after cutting a thin slice from this cube (B). Insert: distribution of the thickness measurements.

Appendix C

WATER FRACTION IN FOILS

The results obtained in Figure 8 are broadly consistent with the theoretical model of laminar diffusion described by Stejskal and Tanner. However, certain deviations from this model have been observed. To address this, we propose a modification to a modified Stejskal-Tanner theory that accounts for a fraction of water within the polyethylene films. If such a fraction indeed exists, it is likely to consist of water trapped between the pores of the film. This trapped water could inhibit the decrease in the diffusion signal, as the molecules cannot move and therefore do not experience phase separation. Consequently, this would lead to an enhancement of the diffusion signal, which deviates from the theoretical expectations. To incorporate this effect, we introduce a modified equation that includes this correction.

$$R_f = \frac{RL + dc}{L + dc}, \tag{9a}$$

where d is the thickness of polyethylene foil, c is the water concentration in foils and R is the same as described in equation 8. The first term of this equation refers to the contribution of the water layer between the polyethylene foils to the total signal, while the second term represents the contribution from the water molecules trapped inside the foils. This adjustment provides a more accurate description of the diffusion process in materials where water is trapped in the structure, offering a better fit to the experimental data.

The foil samples were analyzed using FT-IR Infrared Spectroscopy and Thermal Analysis (TGA/DSC).

1. Spectroscopic Analysis – FT-IR Infrared Spectroscopy

The analysis was performed using a Thermo Scientific Nicolet 6700 FTIR spectrophotometer with a diamond ATR (Attenuated Total Reflectance) accessory (Figure 13).

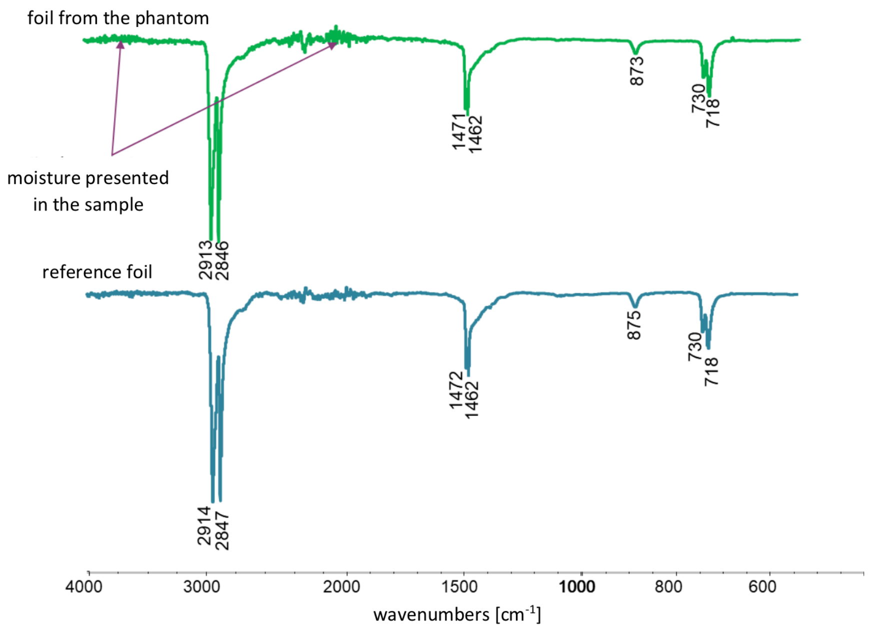

Figure 13. The spectra of the reference PE foil immersed in water just before the analysis and dried with a paper towel (blue color) and the PE foil extracted from the phantom, which had been in water for a long period (green color).

In the spectra of the reference polyethylene foil, signals characteristic of aliphatic C-H bond vibrations are present: symmetric and asymmetric stretching (2914 and 2847 cm^{-1}) as well as symmetric and asymmetric

bending (1472 and 1462 cm^{-1}). Other signals in the spectrum appear in the so-called fingerprint region and are associated with skeletal vibrations (875, 730, and 718 cm^{-1}). No signals related to the vibrations of the amine group bonds (an additive in the foil) were observed due to its low content (below 10%).

In the spectra of the polyethylene foil from water, similar to the reference foil, characteristic signals of aliphatic C-H bond vibrations were observed: symmetric and asymmetric stretching at 2913 and 2846 cm^{-1}, as well as symmetric and asymmetric bending (1471 and 1462 cm^{-1}). Other signals in the spectrum appear in the fingerprint region and correspond to skeletal vibrations (873, 730, and 718 cm^{-1}). No signals related to the vibrations of the amine group bonds (an additive in the foil) were observed due to its low content (below 10%). Slight shifts in signals compared to the reference sample result from the measurement method used and are not considered significant.

In the range of 4200–4500 cm^{-1} and 2700–2200 cm^{-1}, slight baseline noise was observed, likely caused by moisture in the sample.

2. Thermal Analysis (TGA/DSC)

The thermogravimetric analysis (TGA) and differential scanning calorimetry (DSC) were performed using a Mettler Toledo Star TGA/DSC thermogravimetric analyzer (Figure 14,15).

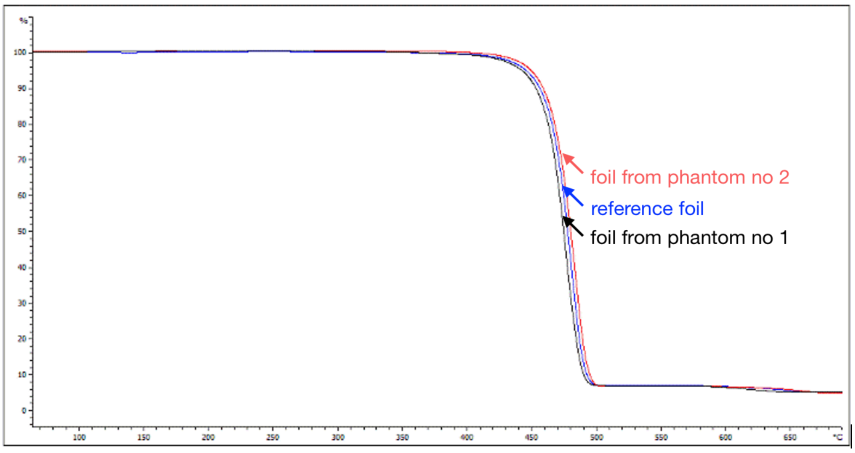

Figure 14. TG thermograms of PE foils.

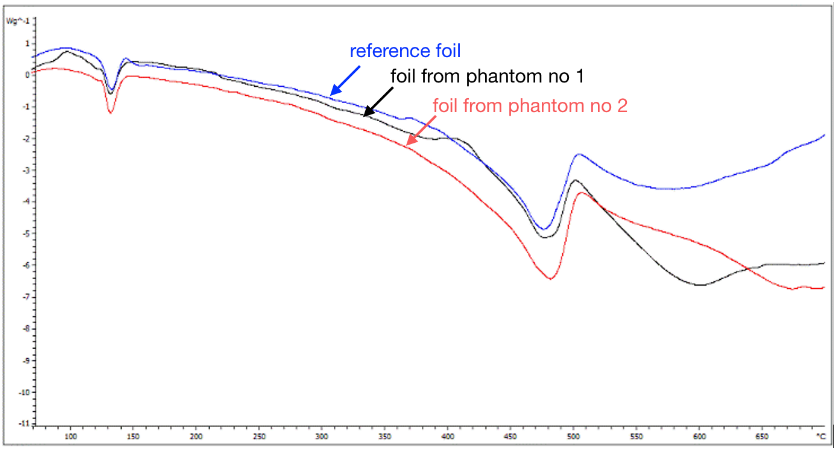

Figure 15. DSC thermograms of PE foils.

Thermal analyses were conducted on untreated PE foil (reference foil) and two samples of foil removed from water and dried with a lint-free cellulose wipe (no 1 and no 2). Approximately 3 mg of each sample

was placed in an alumina crucible and heated in the temperature range of 50-700 °C (temperature ramp rate: 10 °C/min). The analysis was conducted in an inert gas atmosphere -Ar.

The mass loss in the range of 400-650 °C is associated with the thermal decomposition of the foil (Figure 14). No mass loss was observed in the 50-200 °C range related to the removal of water (moisture) from the samples.

In Figure 15 (DSC), endothermic peaks are visible in the range of 124-140 °C, corresponding to the phase transition of PE (the melting temperature of the crystalline phase of the foil). Endothermic peaks in the range of 350-650 °C are associated with the thermal decomposition of the foil.

The presence of such a water fraction in the polyethylene layers was not confirmed by analytical methods such as infrared spectrophotometry and thermogravimetric analysis. Moreover, scanning electron microscopy did not reveal any pores in which water could be trapped (Fig. 16). Additionally, no differences were observed between the reference foil directly from the manufacturer and the foil extracted from the phantom—both exhibited the same surface structure in SEM images. On the samples taken from water, only an increased number of 'artifacts' on the foil surface were observed, likely contaminants that accumulated during the sample preparation process.

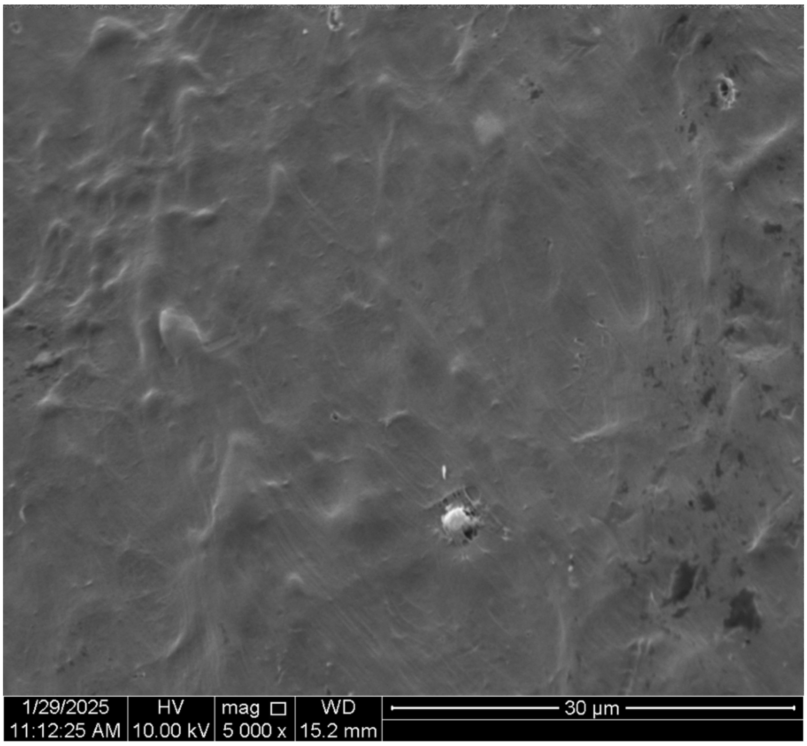

Figure 16. Image of the polyethylene foil from a scanning electron microscope (TFP 2017/12, Inspect S50, FEI). Gold coating was applied using a Leica sputter coater. Magnification: 5000x.

Taking all of this into account, the water fraction is not the cause of the discrepancies observed in Figure 7, and another model is needed.
